# Imaging of current density distributions with a Nb weak-link scanning nano-SQUID microscope

**DOI:** 10.1038/srep15097

**Published:** 2015-10-13

**Authors:** Yusuke Shibata, Shintaro Nomura, Hiromi Kashiwaya, Satoshi Kashiwaya, Ryosuke Ishiguro, Hideaki Takayanagi

**Affiliations:** 1 Division of Physics, University of Tsukuba, Tennodai, Tsukuba, 305-8571, Japan; 2 National Institute of Advanced Industrial Science and Technology (AIST), Umezono, Tsukuba, 305-8568, Japan; 3 Department of Mathematical and Physical Sciences, Faculty of Science, Japan Women’s University, Mejirodai, Bunkyo-ku, Tokyo 112-8681, Japan; 4 Department of Applied Physics, Faculty of Science, Tokyo University of Science, Niijuku, Katsushika-ku, Tokyo 125-8585, Japan

## Abstract

Superconducting quantum interference devices (SQUIDs) are accepted as one of the highest magnetic field sensitive probes. There are increasing demands to image local magnetic fields to explore spin properties and current density distributions in a two-dimensional layer of semiconductors or superconductors. Nano-SQUIDs have recently attracting much interest for high spatial resolution measurements in nanometer-scale samples. Whereas weak-link Dayem Josephson junction nano-SQUIDs are suitable to miniaturization, hysteresis in current-voltage (*I-V*) characteristics that is often observed in Dayem Josephson junction is not desirable for a scanning microscope. Here we report on our development of a weak-link nano-SQUIDs scanning microscope with small hysteresis in *I*-*V* curve and on reconstructions of two-dimensional current density vector in two-dimensional electron gas from measured magnetic field.

Superconducting quantum interference devices (SQUIDs) utilize phase difference of two-weakly coupled superconducting electrodes with Josephson junctions or weak links forming a superconducting loop^1^. The supercurrent flowing in a SQUID is a periodical function of the flux penetrating the superconducting loop Φ divided by the magnetic flux quantum 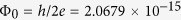 Wb. SQUIDs have been widely used as one of the most sensitive magnetic sensors^2^,^3^,^4^,^5^,^6^. Images of magnetic flux are captured by a SQUID probe scanned on the surface of a sample^2^,^4^. Since there has been increasing interests in detecting magnetic field of small area in order to characterize nanostructures or nano-devices, nano-meter scale SQUIDs have been actively studied recently. Decreasing the size of the superconducting loop favours sensitivity to local magnetic dipoles due to decrease in the limiting flux spectral density^3^ and due to reduced distance between magnetic dipoles and the SQUID loop^7^,^8^. The size of the superconducting loop was typically 2–100 *μ*m^2^,^4^,^9^. Recently, a self-aligned SQUID has been fabricated on a tip of a sharpened quartz tube in the range of 40–300 nm in diameter and has been used for scanning probe magnetometry^6^. This method has the highest spatial resolution to date, but the high flexibility of the design and configuration of SQUIDs makes weak-link nano-SQUIDs fabricated by a focused ion beam (FIB) process^10^,^11^ attractive for use as local probe of magnetic flux.

Weak-link superconducting junctions are fabricated by direct milling using Ga^ + ^ions without lithography process. Simultaneous observations of milled area enable us to precisely position the SQUID and control the properties of the weak-link superconducting junctions^10^,^11^. This is especially advantageous to fabricate a weak-link nano-SQUID on a tip of a scanning probe. Multiple SQUIDs may be fabricated on a scanning probe by FIB process, which may find applications for mappings of magnetic field vector. Weak-link SQUIDs have a number of advantages over tunnel junction based SQUIDs. Since the size of weak-link junctions can typically be made smaller than the size of tunnel junction, the capacitance and the inductance are smaller in weak-link junctions than those in tunnel junction based SQUIDs. This increases sensitivity to magnetic flux and tolerance for high magnetic field environments. However, the presence of hysteresis in current-voltage (*I*-*V*) characteristics^10^ has been an obstruct for a weak-link nano-SQUID to be used in scanning probe measurements.

In this paper, we describe construction and characterization of our weak-link scanning nano-SQUID microscope (Fig. 1(a–e)) with small hysteresis in *I*-*V* characteristics suitable for imaging. We present results of imaging of magnetic field created by current in a Hall-bar structure of a GaAs/Al_0.3_Ga_0.7_As modulation-doped single heterojunction to evaluate performance of the weak-link scanning nano-SQUID microscope, and report on reconstruction of the two-dimensional current density vector by a Fourier analysis.

## Fabrication of weak-link nano-SQUID probes

**Figure 1:**
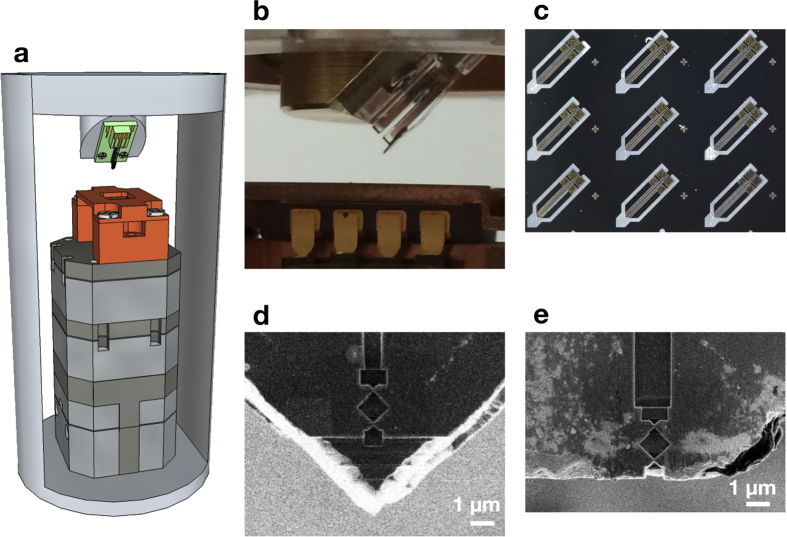
Schematics of scanning nano-SQUID system and images of nano-SQUID probes. (**a**) Schematic illustration of a scanning nano-SQUID system in a cryogen-free ^4^He refrigerator. Upper green part indicates a holder for a scanning SQUID probe attached to a quartz tuning fork. A sample stage is placed on triaxial piezoelectric inertial stages. The SQUID probe and the piezoelectric inertial stages are settled in a stainless housing which is connected to the 4 K plate with four springs. (**b**) Optical image of a SQUID probe attached to a tuning fork and a sample chip carrier. (**c**) Optical image of probes fabricated by a laser-lithography and deep etching of a silicon substrate. After detaching each pieces, a nano-SQUID was fabricated at the tip of the probe by an FIB. (**d**) Scanning ion microscope images of SQUID probes without mechanical polishing and (**e**) after mechanical polishing of the tip of the probe. SQUID loop and weak link junctions were fabricated by an FIB milling system.

Nb/Au thin films were deposited on a Si substrate. Nb film with the thickness of 23 nm was deposited by an RF-sputtering and subsequently Au film with the thickness of 70 nm was deposited by an electron-beam deposition at the base pressure of 6 × 10^6^ Pa. The Au thin film was used to protect the Nb film from Ga^+^ ion beam exposure. The Au thin film was also important to reduce the hysteresis in the *I*-*V* curve of a nano-SQUID by reducing the self-heating effect at the weak-link junctions^10^,^12^. Superconducting four-terminal configurations were patterned by a maskless laser lithography using 405 nm light (DL-1000, NanoSystem Solution, inc). Silicon probes for scanning miscroscopy with a thickness of 100 *μ*m and the size of 2000 × 600 *μ*m^2^ were defined by a maskless laser lithography. Deep etching of silicon substrate with high aspect ratio was performed by 200 cycles of a pulsed reactive ion etching (RIE) by SF_6_ and deposition of a C_4_F_8_ passivation layer. About a hundred pieces of Si probes were fabricated by the above process. (Fig. 1(c)) The tip of a part of Si probes was mechanically polished (Supplementary Fig. 1, and Supplementary Note 1) to remove Nb/Au film near the edge damaged by the RIE process using Al_2_O_3_ powder with an average diameter of 100 nm dispersed in 1-octanol. Each Si probe was cut into a piece. A Si probe was mounted on a quartz tuning fork with a resonance frequency of 32,768 Hz. After gluing the Si probe to a quartz tuning fork, the resonance frequency decreases to typically 30.8 KHz with the Q-factor of about 10,000 at 4 K. Finally, the superconducting loop and weak-link junctions were milled by the FIB process with typical beam voltage and current of 31 kV and 5 pA, respectively. The SQUID loop with the size of 1 *μ*m was located within 4.8 *μ*m from the tip of the Si probe. The dimensions of the SQUID loop and the weak-link width were 1.0 *μ*m and 80 nm, respectively. (Fig. 1(d,e)) The geometrical inductance 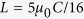, where *C* is the inner circumference of the SQUID^13^ was 1.6 pH. The effect of the self-induced field as given by 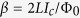 was 0.32.

## Results and Discussion

### Characteristics of SQUID probes

**Figure 2:**
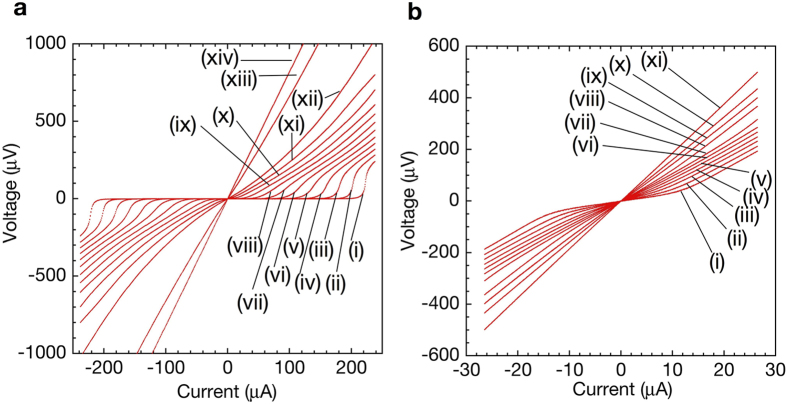
Temperature dependence of current-voltage characteristics of SQUID probes. (**a**) Current-voltage characteristics of a SQUID probe without mechanical polishing at *T* = (i) 3.6, (ii) 3.8. (iii) 4.0, (iv) 4.2, (v) 5.0, (vi) 5.2, (vii) 5.4, (viii) 5.6, (ix) 5.8, (x) 6.0, (xi) 6.2, (xii) 6.4, (xiii) 6.6, (xiv) 6.8 K. The current was swept for both negative and positive directions, and no hysteresis in the *I*-*V* characteristics was observed. (**b**) Current-voltage characteristics of a SQUID probe after mechanical polishing at *T* = (i) 3.6, (ii) 3.8. (iii) 4.0, (iv) 4.2, (v) 5.0, (vi) 5.2, (vii) 5.4, (viii) 5.6, (ix) 5.8, (x) 6.0, (xi) 6.2 K. No hysteresis in the *I*-*V* characteristics was observed.

Figure 2(a,b) show *I*-*V* characteristics of nano-SQUID probes without and after mechanical polishing, respectively, at temperatures between *T* = 3.6 and 6.8 K at zero applied magnetic field. The current was swept in both negative and positive directions, and no hysteresis was observed in the *I*-*V* characteristics in Fig. 2(a,b). The Dayem nano-SQUID was shown to exhibit a hysteretic behaviour for 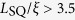
14, where *L*_SQ_ is the Dayem nano bridge size and 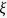 is the Ginzburg-Landau coherence length, which is 38 nm for Nb^1^. The constrictions of the nano-SQUID were milled to wedged shape by the FIB process with the weak link width of *w* = 80 nm. Although the model by Likharev^14^ is not directly applicable to the geometry of our nano-SQUIDs, the nonhysteretic behaviour of *I*-*V* characteristics is explained by small 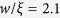. Furthermore, we reduced the effects of Joule heating by reducing the critical current *I*_*c*_ and preparing Au film above Nb film for heat conduction, which is also important for the nonhysteretic behaviour of *I*-*V* characteristics of our nano-SQUIDs.

**Figure 3:**
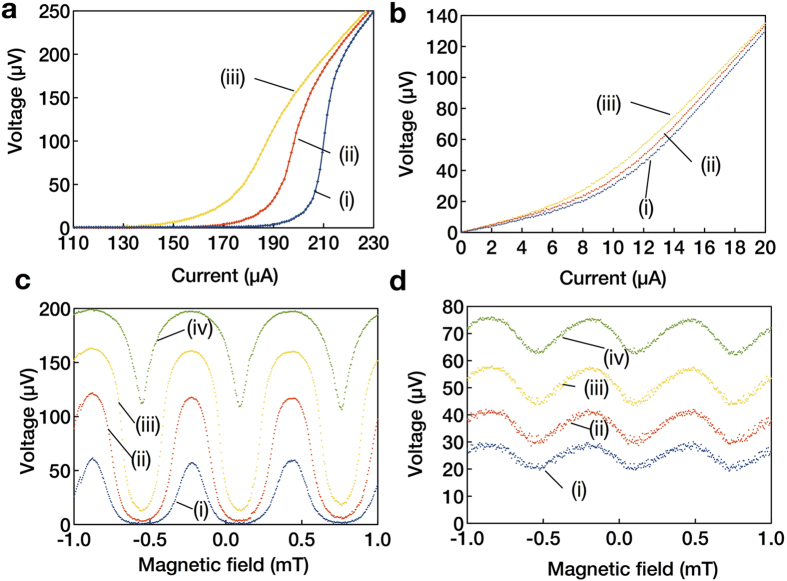
Magnetic field dependence of current-voltage characteristics of SQUID probes and modulation of the SQUID voltage by magnetic field. (**a**) Typical current-voltage characteristics of a nano-SQUID probe without mechanical polishing and (**b**) after mechanical polishing of the tip of the probe at applied magnetic field of (i) 0.5, (ii) 0.3, and (iii) 0.1 mT. No hysteresis in the *I*-*V* characteristics was observed. (**c**) Modulation of SQUID voltage (*V*_SQ_) by *B*_ext_ at current-bias of (i) 196.2, (ii) 198.1, (iii) 200.0, and (vi) 201.9 *μ*A for a nano-SQUID probe without mechanical polishing. (**d**) Modulation of SQUID Voltage (*V*_SQ_) in *B*_ext_ at current-bias of (i) 8.0, (ii) 10.0, (iii) 12.0, and (vi) 14.0 *μ*A for a nano-SQUID probe after mechanical polishing.

The critical temperature *T*_*c*_ of the nano-SQUID without mechanical polishing was 6.0 K, which is reduced from typical *T*_*c*_ of Nb of 9.2 K due to small thickness of the Nb film and the proximity effect between Nb and Au^10^. Figure 3(a) shows a typical *I*-*V* characteristics of the nano-SQUID probe without mechanical polishing (Fig. 1(d)) at applied magnetic fields between 0.1 and 0.5 mT at 4.2 K. The critical current *I*_*c*_ is seen to change with the applied magnetic field.

The SQUID loop of the probe after mechanical polishing of the tip of the nano-SQUID probe (Fig. 1(e)) was located very close to the edge of the Si substrate and the probe did not exhibit zero resistance as shown in Fig. 2(b) above 3.6 K because of the damage of Nb film during the fabrication process. Nevertheless, the change of the voltage with the applied magnetic field was observed as shown in Fig. 3(b), indicating that this nano-SQUID probe is useful as a magnetic field sensor. The nonhysteretic behaviour of our nano-SQUIDs enables us to measure the voltage of a nano-SQUID at a constant bias current to obtain magnetic flux threading the SQUID loop. This is particularly advantageous to use a nano-SQUID as a magnetic sensor for a scanning microscope. If a nano-SQUID shows a hysteretic *I*-*V* characteristic, *I*_*c*_ has to be measured by sweeping *I* at each point. Typical voltage-magnetic field (*V*_SQ_ − *B*_ext_) characteristics in Fig. 3(c,d) show oscillations of SQUID voltage *V*_SQ_ with *B*_ext_ at constant current-bias. The noise of the probe without mechanical polishing was estimated to be 3.1 nT/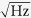 at 2 kHz. The noise of the probe after mechanical polishing was degraded to 40 nT/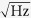 at 2 kHz while the spatial resolution was improved.

### Mapping of magnetic field distribution around Nb/Au strips

**Figure 4:**
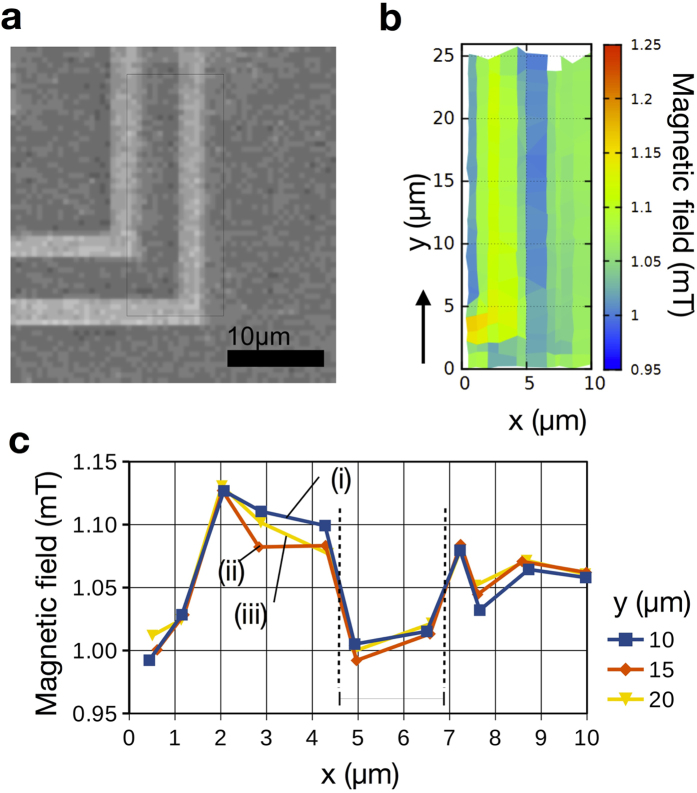
Image and mapping of magnetic field distribution of Nb/Au strips. (**a**) Scanning electron microscope image of Nb/Au strips with a width of 2 *μ*m and a spacing of 5 *μ*m at applied magnetic field of 1.05 mT at 4 K. (**b**) Mapping of magnetic field distribution of Nb/Au strips. (**c**) Line profiles of magnetic field distribution along (i) *y* = 10, (ii) 15, and (iii) 20 *μ*m.

Mapping of magnetic field distribution around Nb/Au strips was performed to test spatial resolution of our nano-SQUID microscope. The thicknesses of Nb and Au films were 600 and 30 nm, respectively. The width and the spacing of Nb/Au strips were 2 and 5 *μ*m, respectively, as shown in Fig. 4(a). A nano-SQUID probe after mechanical polishing was used at a constant distance between the tip of the SQUID probe and the sample surface of 1.2 *μ*m. The obtained mapping of magnetic field distribution of Nb/Au strips at an applied magnetic field of 1.05 mT is shown in Fig. 4(b). A strip of reduced magnetic field because of the Meissner effect of the Nb strips can be identified at 5 < *x* < 7 *μ*m. The reduction of magnetic field is more clearly seen in the lineprofiles of magnetic field along *y* = 10, 15, and 20 *μ*m as shown in Fig. 4(c), indicating that the spatial resolution of our nano-SQUID microscope is better than 2 *μ*m.

### Mappings of magnetic field distribution induced by current in a modulation-doped single heterojunction sample

**Figure 5:**
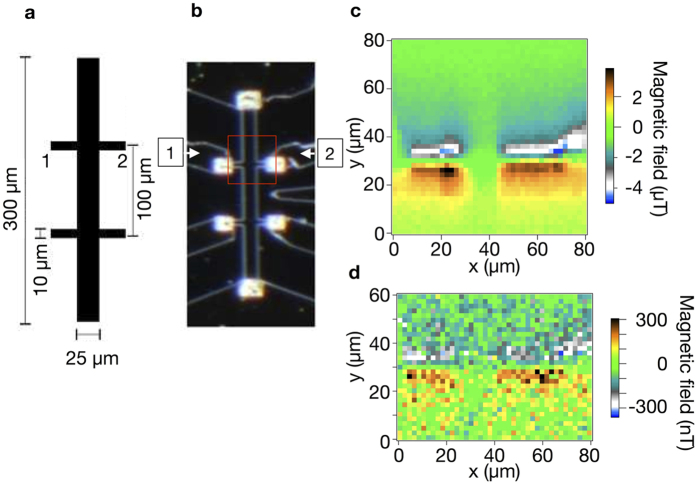
Hall-bar structure and mappings of magnetic field distribution by the current flowing in the Hall-bar sample. (**a**) Schematic structure of a sample Hall-bar. (**b**) Optical micrograph of a sample Hall-bar structure. Red square indicates the scanning area of 80 *μ*m × 80 *μ*m for (**c**). Mappings of magnetic field distribution induced by current in a GaAs/Al_*x*_Ga_1−*x*_As modulation-doped single heterojunction sample at *T* = 4 K for current (c) 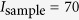 and (d) 2.8 *μ*A at the applied magnetic field by a superconducting magnet of 0.0 mT using a nano-SQUID probe after mechanical polishing of the tip of the probe. The height of the SQUID probe from the sample surface was 1.5 *μ*m.

Results of mappings of magnetic field created by ac current between the voltage probes 1 and 2 of the Hall-bar structure (Fig. 5(a,b)) at 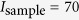 and 2.8 *μ*A are shown in Fig. 5(c,d), respectively, at *T* = 4 K at the applied magnetic field by a superconducting magnet of 0.0 mT using a probe after mechanical polishing. The measurements were performed in unshielded laboratory environment, and hence the sample and the nano-SQUID probe were subject to the environmental magnetic field. The size of the scanning area was 80 × 80 *μ*m^2^ with a step size of 2 *μ*m for *x*- and *y*-directions. The height of the SQUID probe from the sample surface was *z*_0_ = 1.5 *μ*m. The voltage of a SQUID at a constant current bias of 12 *μ*A changes due to magnetic field created by the current in the Hall-bar structure. This voltage change was synchronously detected with a lock-in amplifier at a time constant of 1 s by scanning the position of the nano-SQUID probe on the surface of the sample. The bias current of 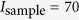 and 2.8 *μ*A corresponds to current density of 7.0 and 0.28 A/m, respectively. The mapping in Fig. 5(c) (Fig. 5(d)) corresponds to the case of the current density above (below) the condition for the breakdown of the quantum Hall effect^15^,^16^. The maximum source-drain voltage (*V*_SD_) was 8.0 mV at 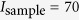 *μ*A. The Fermi energy of the electrons is 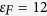 meV at the electron density of the sample of 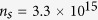 m^−2^, which gives 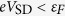. The positive and negative magnetic fields are observed near the edges of the stem of the Hall-bar structure with the width of 10 *μ*m as shown in Fig. 5(c). The magnetic field distributions are seen to be broadened in the center region where the 10 *μ*m width stem crosses with the bar with the width of 25 *μ*m. Similar structures are seen in the magnetic field distribution at 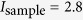 *μ*A (Fig. 5(d)) although the signal-to-noise ratio was degraded.

### Reconstruction of two-dimensional current density distribution

Two-dimensional current density distribution **J**(*x*, *y*) can be reconstructed from the measured magnetic flux distribution **B**(*x*, *y*, *z*) based on a Fourier analysis^17^. The two-dimensional Fourier transform of the current density and magnetic field is defined by


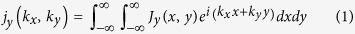


and


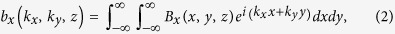


respectively. By using the two-dimensional Fourier transform of the Green’s function


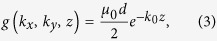


we have


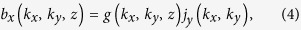


where *d* is the thickness of the current and we defined 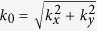. Similarly, *b*_*z*_ is given by


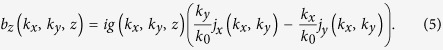


We assume quasi-stationary current density 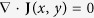 in real-space and 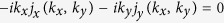 in *k*-space. A nano-SQUID probe facing *θ* to the surface of the sample detects 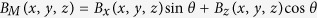. Because a nano-SQUID detects magnetic field averaged over the square SQUID loop, the Fourier transform of measured magnetic field 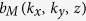 should be divided by^17^


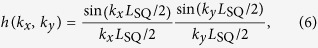


where *L*_SQ_ is the size of the SQUID loop to take into account the finite size of the SQUID in *x*- and *y*-directions. In the case of our nano-SQUID, 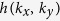 does not introduce noticeable difference because *L*_SQ_ is smaller than the step size of the measurement. For the *z*-direction, we assume a SQUID probe detects the average magnetic field *B*_ave_ at the effective height *z*_eff_ satisfying


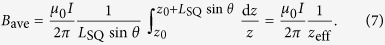


For *z*_0_ = 1.5 *μ*m, *L*_SQ_ = 1.0 *μ*m, and *θ* = 51°, we obtain *z*_eff_ = 1.9 *μ*m.

We substitute 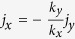, into equation (5), and using equation (4), we obtain


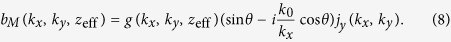


The current density can be readily obtained as


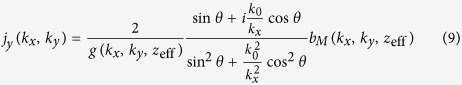


in *k*-space and


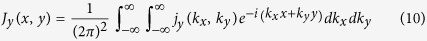


in real-space. 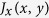 may be similarly obtained by using 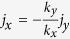. For the case of 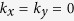, *j*_*x*_(0, 0) and *j*_*y*_(0, 0) cannot be determined from 
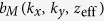
. The obtained *J*_*x*_ and *J*_*y*_ have ambiguity by uniform current density distribution. In the following analysis, we set this uniform current density as zero.

**Figure 6:**
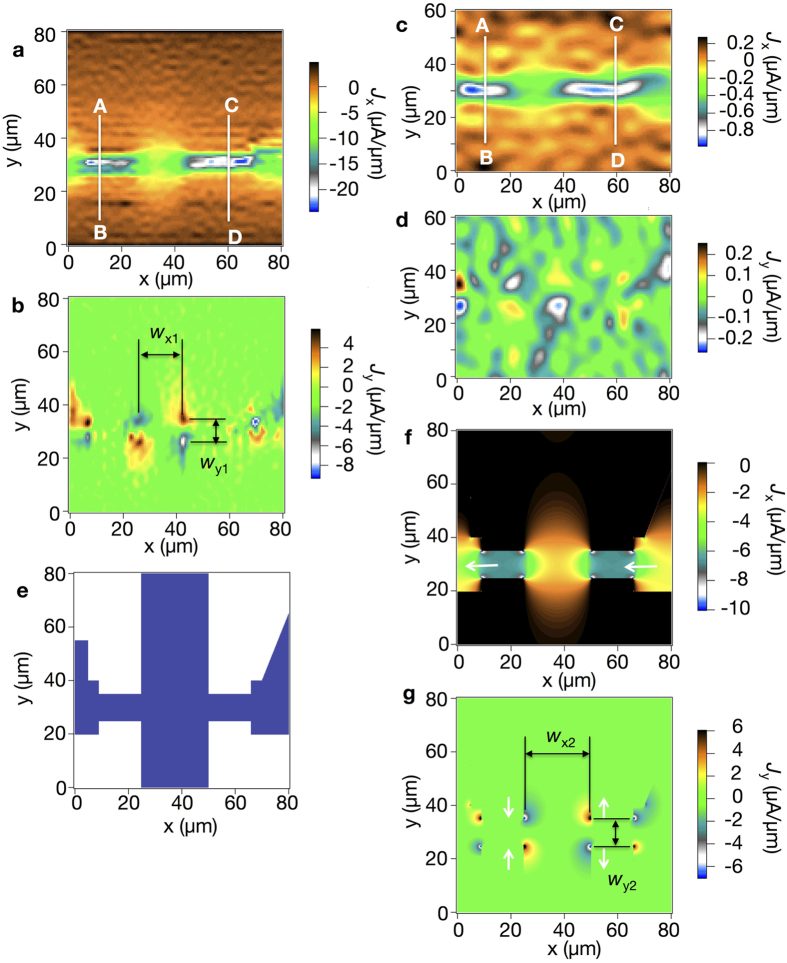
Reconstructed current density distributions and calculated current densities. (**a**) Reconstructed current density distributions 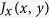 and (**b**) 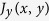 from the measured magnetic flux in Fig. 5 at 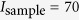 *μ*A. (**c**) 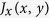 and (**d**) 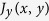 reconstructed from the measured magnetic flux in Fig. 5 at 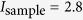 *μ*A. (**e**) Pattern of Hall-bar structure assumed for *σ*(*x*, *y*). Blue and white indicate area with 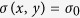 and 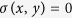, respectively. (**f**) Calculated current density 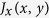 and (**g**) 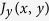, assuming isotropic conductivity 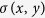. The white arrows indicate the direction of the current.

Figure 6(a–d) show reconstructed current density distributions 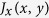 and 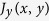 from the measured magnetic flux in Fig. 3(b,c) at 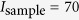 and 2.8 *μ*A, respectively, for *θ* = 51°. Calculations were performed on a mesh of 128 × 128 in the *x* and *y* directions. A Parzen window^17^,^18^


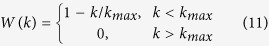


was used to eliminate high-spatial-frequency components of measured mappings^17^. We empirically chose 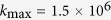 and 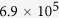 m^−1^ for 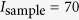 and 2.8 *μ*A, respectively, so that high-spatial-frequency noise is effectively reduced with minimum loss of spatial resolution. In the center region, where the 10 *μ*m-stem crosses the bar with the width of 25 *μ*m, the current density 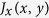 is seen to spread to the wider bar. This can be more clearly indicated in 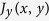 in Fig. 6(b) by the positive and negative current density near the corners of the mesa structure of the Hall-bar. At smaller current of 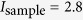 *μ*A, the main features of the current densities can still be resolved as shown in Fig. 6(c,d). Although the signal-to-noise ratio of the mapping of magnetic field in Fig. 5(d) is heavily degraded as compared to the case of 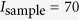 *μ*A in Fig. 5(c), the current densities are recovered at the expense of degraded spatial resolution by using 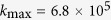 m^−1^ used for the Parzen window. The mappings of the magnetic field (Fig. 5(c)) and the reconstructed current density distributions (Fig. 6(a,b)) using the nano-SQUID probe after mechanical polishing show remarkably better spatial resolution as compared to the mappings using the nano-SQUID probe without mechanical polishing (Supplementary Fig. 2). This shows the advantage of the nano-SQUID probe after mechanical polishing over the unpolished nano-SQUID probe although the zero resistance was not observed due to the damage of Nb film in the polished nano-SQUID probe. The current obtained by integrating current densities 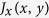 on the white bars in Fig. 6(a) is 84 and 91 *μ*A for the crosssection A-B and C-D, respectively, in reasonable agreement with 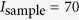 *μ*A. Similarly, current across the white bars in Fig. 6(c) is 4.5 and 4.6 *μ*A for the crosssection A-B and C-D, respectively, for 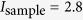 *μ*A.

### Comparison of the measured current density with results of a numerical calculation

Two-dimensional quasi-stationary current density is calculated by assuming that the current density instantaneously responds to the electric field at the position of the electron as given by^19^


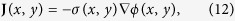


which is applicable to a low mobility limiting case. Clearly, this model is not directly applicable to high mobility electron gas, nonetheless this model is useful to assist the understandings of the observed current density mappings by our SQUID microscope. One may refer to semiclassical^20^,^21^ or quantum mechanical^22^ ballistic electron transport theories for more realistic descriptions. The measured current density **J**(*x*, *y*) may be described by the statistical or quantum mechanical average of the local electric current operator, which is described by the momentum **p** and the position **x** of an electron. Then ballistic electron transport theories take into account the scatterings of the electron from **p** to 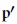 by such as the external electric field, the impurities, and the phonons. The description implied by equation (12) ignores these effects, in particular, the inertial motion of the current carrying electrons.

We also assume slow modulation frequency of the current with negligible displacement current 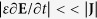 and isotropic conductivity 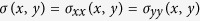. Then we solve numerically


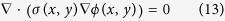


by a finite-difference method on a mesh of 400 × 400 with *σ*_0_ = 0.05 S, where *ϕ*(*x*, *y*) is the electrostatic potential. Conductivity was assumed to be 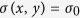 inside the Hall bar structure and 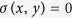 elsewhere as shown in Fig. 6(e). Dirichlet boundary conditions were applied for *x*-direction, and the voltage was set to be −0.798 and 0 meV at *x* = 0 and 80 *μ*m, respectively. A periodic boundary condition was applied to the *y*-direction. The electrostatic potential *ϕ*(*x*, *y*) was converged to an accuracy of less than 10^−6^ V.

Figure 6(f,g) show calculated current density. The observed main features in Fig. 6(a,b) are reproduced in Fig. 6(f,g). In particular, the spread of 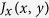 to the wider stem of the Hall-bar in Fig. 6(a) is reproduced in Fig. 6(f), and the local maxima and minima in 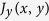 near the corners of the mesa structure of the Hall-bar in Fig. 6(b) are reproduced in Fig. 6(g). The distances between the local maxima and minima in 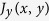 are 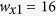 *μ*m in Fig. 6(b) and 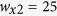 *μ*m in Fig. 6(g), indicating a sizable disagreement with 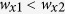, whereas the difference in 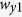 in Fig. 6(b) and 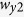 in Fig. 6(g) is small. This disagreement is not explained by the finite *k*_*max*_ for the truncation of high-frequency components in the Fourier analysis. We calculated the magnetic field due to the calculated current density as given by equation (12), and reconstructed the current density distributions by changing *k*_*max*_ by the method described by equations (1)-(11), 17, 18, 19, 21, 26, 28, 30, 31, 32, 40 and checked that 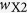 and 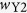 did not depend on *k*_*max*_. Consequently the disagreement between the observed and the calculated distances between the local maxima and minima in 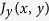 is understood by the finite length that the electrons travel before they change momenta following the direction of the gradient of the electrostatic potential 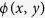. The difference 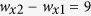 *μ*m is reasonably explained by the mean free path of the sample of 8.7 *μ*m. Thus the disagreement between 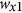 and 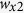 is a manifestation of ballistic transport of the electrons. The reconstructed 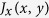 in Fig. 6(a) is nearly symmetric and four main peaks in 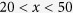 *μ*m in Fig. 6(b) are nearly antisymmetric with respect to the center of the 25 *μ*m-width bar, because ac bias current was applied to the Hall-bar structure and the magnetic field was detected synchronously using a lock-in amplifier. The effect of the limited spatial resolution due to small *k*_max_ is observed in Fig. 6(a) where the current density is larger at the center of the Hall-bar than the edge unlike the case in Fig. 6(f) where the current density is nearly homogeneous across the stem of the Hall-bar with the width of 10 *μ*m. The depletion layer thickness was estimated to be 134 nm^23^,^24^ for similar GaAs single heterojunction sample with the electron density of 4.6 × 10^15^ m^−2^. The height of the mesa structure of the sample was about 100 nm. Thus the depletion layer thickness of the two-dimensional electron gas and the widths of the lateral etching are too small to explain the measured current density distribution. Improvements in the signal-to-noise ratio of the magnetic flux measurements are required to increase *k*_max_ to obtain better spatial resolution.

In summary, we have constructed a weak-link scanning nano-SQUID microscope using a SQUID probe with small hysteresis in *I*-*V* curve suitable for a magnetic sensor for scanning measurements. We have measured magnetic field distribution created by the current in the Hall-bar structure. Two-dimensional current density components 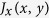 and 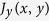 were reconstructed from measured *B* based on a Fourier analysis. The reconstructed two-dimensional current density reproduced most of the features of current density calculated by solving Laplace equation, however, a significant deviation was found near the corners of the Hall-bar structure and was explained by ballistic electron transport. Our newly developed scanning nano-SQUID microscope may contribute to characterize, for example, chiral or helical superconductors and current density in the quantum anomalous Hall effect or in the quantum spin Hall effect.

## Methods

### Low temperature scanning SQUID microscope

A nano-SQUID probe was accommodated in a rigid stainless housing connected with four springs to the 4 K plate of a cryogen-free pulse tube refrigerator (Optistat PT, Oxford Instruments) to reduce vibrations due to cooling cycle. A sample was mounted on closed loop inertially actuated triaxial stepping piezoelectric-stages with resistive position encoders (ANPx101/RES and ANPz101/RES, Attocube systems). The piezoelectric-stages were operated in the coarse positioning mode with typical minimum step size of 10 nm and in the fine positioning mode. The repeatability of the resistive position encoders were estimated to be about 200 nm at 4 K. The stainless housing was placed in the bore of a homemade superconducting magnet with the bore size of 50 mm. Control of the distance between a nano-SQUID probe and the sample surface was made by monitoring the shift of the resonance frequency of a quartz tuning fork using a lock-in amplifier^25^. First, the *z*-axis stage was moved by a step of about 100 nm in the coarse positioning mode. Second, the *z*-axis stage was scanned by monitoring the signal of the quartz tuning fork in the fine positioning mode. This procedure was repeated until the resonance frequency shift of the quartz tuning fork due to the nano-SQUID probe-sample interaction was detected. After detecting the height of the sample surface, scanning of the nano-SQUID probe was performed at a constant height above the sample surface by monitoring the resistive position encoder. The voltage of a SQUID was amplified by a preamplfier (LI-75A, NF Corporation) at room temperature. The measurements were performed in unshielded laboratory environment. Because the size of the SQUID loop is small, the contribution from the environment magnetic field noise is relatively small. The noise of the SQUID probes is mainly limited by the equivalent input noise of the preamplifier.

### Sample

The sample GaAs/Al_0.3_Ga_0.7_As modulation-doped single heterojunction consists of a GaAs/AlAs superlattice buffer layer, 200 nm-thick undoped GaAs layer, 40 nm-thick undoped Al_0.3_Ga_0.7_As layer, 30 nm-thick Si-doped Al_0.3_Ga_0.7_As layer, and 10 nm-thick Si-doped GaAs capping layer. The density and the mobility of the two-dimensional electron gas were 3.3 × 10^15^ m^−2^ and 91 m^2^/Vs at 2.8 K, giving the mean free path of electrons of 8.7 *μ*m. A Hall-bar structure of the width and the length of 25 and 300 *μ*m was fabricated by photolithography. The distance between the voltage probes was 100 *μ*m. Current between ohmic contacts of the Hall-bar structure was modulated at 1873 Hz. The voltage of a SQUID probe at a constant current bias changes due to magnetic field created by the current in the Hall-bar structure. This voltage change was detected synchronously with a lock-in amplifier at a time constant of 1 s by scanning the nano-SQUID probe on the surface of the sample.

## Additional Information

**How to cite this article**: Shibata, Y. *et al.* Imaging of current density distributions with a Nb weak-link scanning nano-SQUID microscope. *Sci. Rep.* **5**, 15097; doi: 10.1038/srep15097 (2015).

## Supplementary Material

Supplementary Information
