# Supplementary material for: Imaging of current density distributions with a Nb weak-link scanning nano-SQUID microscope
Source: Sci Rep. 2015 Oct 13;5:15097. doi: 10.1038/srep15097 (PMC4602221; doi:10.1038/srep15097)
Supplement: Supplementary Information [file srep15097-s1.pdf]

Supplementary Information for Imaging of current density distributions with a Nb weak-link scanning nano-SQUID microscope

Yusuke Shibata[1], Shintaro Nomura[1], Hiromi Kashiwaya[2], Satoshi Kashiwaya[2], Ryosuke Ishiguro[3,4], and Hideaki Takayanagi[4]

[1]Division of Physics, University of Tsukuba, Tennodai, Tsukuba, 305-8571, Japan
[2]National Institute of Advanced Industrial Science and Technology (AIST), Umezono, Tsukuba, 305-8568, Japan
[3]Department of Mathematical and Physical Sciences, Faculty of Science, Japan Women's University, Mejirodai, Bunkyo-ku, Tokyo 112-8681, Japan
[4]Department of Applied Physics, Faculty of Science, Tokyo University of Science, Niijuku, Katsushika-ku, Tokyo 125-8585, Japan

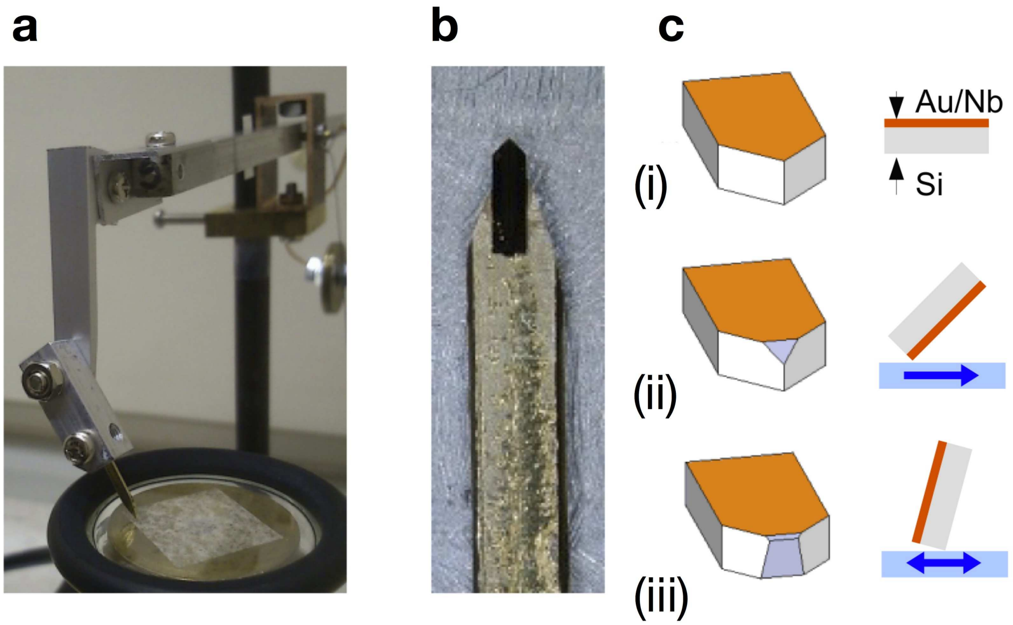

Supplementary Fig. 1: Method of mechanical polishing of a Si probe
(a) Optical photograph of an apparatus for mechanical polishing of a Si probe tip. (b) Optical micrograph of a Si probe tip attached to a brass-supporting rod. (c) Schematics of mechanical polishing method. (i) A Si probe tip before mechanical polishing, (ii) a Si probe tip after first polishing from the topside of the probe, and (iii) a Si probe tip after second polishing of the rear side of the Si probe.

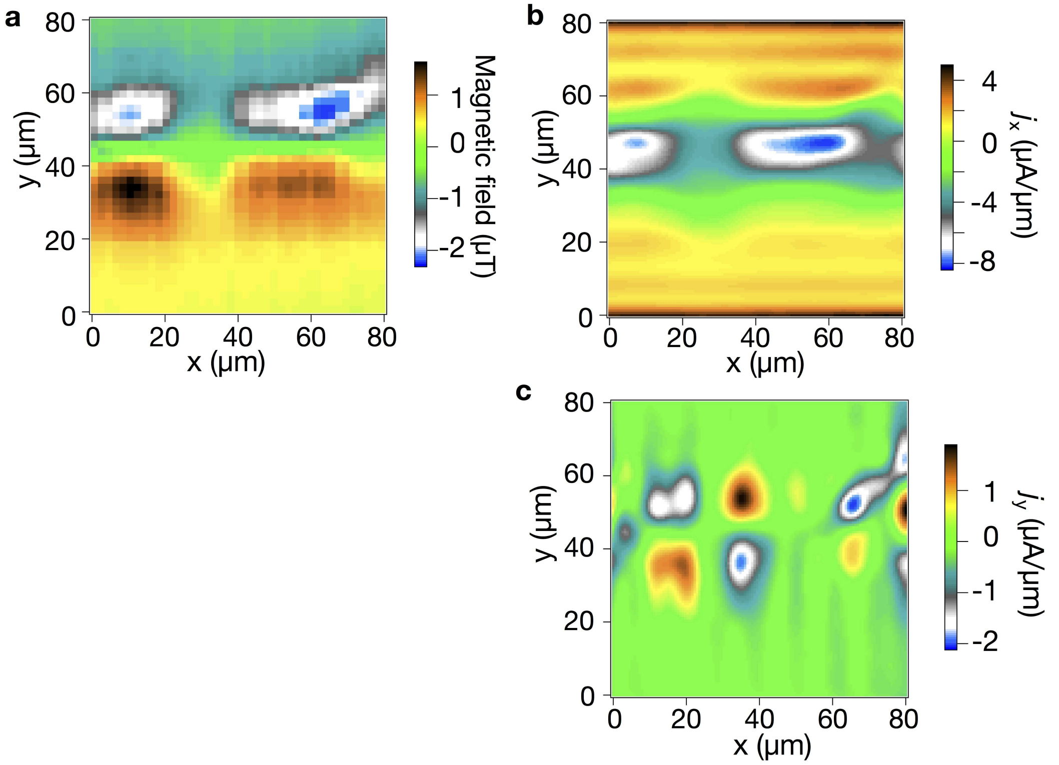

Supplementary Fig. 2: Mappings of magnetic field distribution and reconstructed current density distributions using a nano-SQUID probe without polishing
(a) Mappings of magnetic field distribution induced by current in a GaAs/$Al_xGa_{1-x}As$ modulation-doped single heterojunction sample at $T = 4$ K using a nano-SQUID probe without mechanical polishing, at the applied magnetic field by a superconducting magnet of 0.25 mT. The current was $I_{\mathrm{sample}} = 70\ \mu$A and the height of the SQUID probe from the sample surface was 4.0 μm. (b) Reconstructed current density distributions $J_x(x,y)$ and (c) $J_y(x,y)$ from the measured magnetic flux in (a) using $k_{\mathrm{max}} = 6.0 \times 10^5\ \mathrm{m}^{-1}$ for the Parzen window.

Supplementary Note 1. Technical details of mechanical polishing of Si probe tip

The Nb superconductor film near the tip of the Si probe is damaged during the fabrication process, in particular in silicon deep etching process, suffering the performance of the SQUID. We, therefore, removed damaged part of the Nb film by a mechanical polishing of the tip of Si probes.

Most of commercially available slurries contain water and chemical compounds that suffer superconducting property of Nb film due to oxidation. In order to prevent exposure of the Nb film to water, we prepared slurry using high purity alumina particles (TM-DAR, Taimei Chemicals) with average particle diameter of 100 nm dispersed in n-octanol (Wako Chemicals). The mass ratio of alumina particles and n-octanol was 1:2. The mixture was immersed in a supersonic bath cleaner for about 1 hour to disperse alumina particles. Because the degradation area was several micrometer from the probe substrate edge, precise polishing with sub-micrometer range was important. In order to obtain a constant load during polishing and a fixed angle between the probe tip and the base microscope slide, we constructed an apparatus as shown in Supplementary Fig. 1(a). The Si probe was glued to a brass-supporting rod with two-component epoxy adhesive as shown in Supplementary Fig. 1(b). A constant load for the polishing was achieved by the long supporting rod utilizing a seesaw mechanism. The load was typically 0.4 g, which was calibrated by a digital weighing machine.

By using this apparatus, we polished the edge of Si probes manually. After putting a few drop of slurry on a slid glass, a Si probe was mounted on the drop. The microscope slide was slowly moved manually at a fixed direction. Schematic illustrations of a typical polishing process are shown in Supplementary Fig. 1(c). Supplementary Fig. 1 (c) (i) shows a Si probe with a Nb/Au layer on top before polishing process. First, we removed the degradation area by polishing from the topside of the Si probe (ii). The direction of the polishing was kept to be one-way as shown in Supplementary Fig. 1(c)-(ii) to prevent turning-up of the edge of the Nb/Au film. (The blue arrow indicates the direction of the polishing) The angle of the microscope slide and the Si probe was 45°. The Si probe was slid on the microscope slide for less than 30 mm. We found this short sliding length was enough to polish the tip of Si probe by several microns. The Si probe was cleaned in isopropanol for 10 min in a supersonic bath cleaner, and was inspected by an optical microscope with a x100 objective lens.

In the second polishing process, the rear side of the Si probe was polished as shown in Supplementary Fig. 1 (c)-(iii). We found that the rate of the polishing decreases with the increase in the area where the Si probe touched to the microscope slide. Thus we carefully polished the rear side of the Si probe by repeatedly inspecting the shape of the Si probe using an optical microscope.
